# Pendulum Therapy of Molar Distalization in Mixed Dentition

**DOI:** 10.5005/jp-journals-10005-1336

**Published:** 2016-04-22

**Authors:** Raju Umaji Patil, Amit Prakash, Anshu Agarwal

**Affiliations:** 1 Professor and Head, Department of Pedodontics and Preventive Dentistry Department of Pedodontics and Preventive Dentistry, STES Sinhgad Dental College and Hospital, Pune, Maharashtra, India; 2 Reader, Department of Orthodontics and Dentofacial Orthopedics Rishiraj College of Dental Sciences and Research Centre Bhopal, Madhya Pradesh, India; 3 Reader, Department of Orthodontics and Dentofacial Orthopedics Rishiraj College of Dental Sciences and Research Centre Bhopal, Madhya Pradesh, India

**Keywords:** Class II malocclusion, Distalization, Mixed dentition, Pendulum, Space regainer.

## Abstract

Early and timely pedo-orthodontic treatment is aimed at eliminating the disturbances of skeletal or dentoalveolar development, to harmonize the stomatognathic system before the full eruption of all permanent teeth. The advantages of pendulum appliance are its minimal dependence on patient’s compliance (child cooperation), ease of fabrication, onetime activation and adjustment of the springs if necessary to correct minor transverse and vertical molar positions. This article reports a successful treatment method of class II malocclusion with pendulum appliance in mixed dentition phase. Distalization of maxillary molar was done, followed by guidance of canine impaction orthodontically and other dental correction using 0.022 MBT appliances. Posttreatment results were stable and remarkable.

**How to cite this article:** Patil RU, Prakash A, Agarwal A. Pendulum Therapy of Molar Distalization in Mixed Dentition. Int J Clin Pediatr Dent 2016;9(1):67-73.

## INTRODUCTION

Since all children do not cooperate for dental treatment, it is difficult to manage problems of developing dentition in children with interceptive orthodontic appliances. Pendulum is unique and different as it is a child-friendly appliance. This is why clinicians often prefer intraoral distalization appliances that minimize the need for patient cooperation. Correction of class II malocclusion without extraction requires maxillary molar distalization by means of intraoral or extraoral forces.^1^ For cases with minimal arch length discrepancy and mild class II molar relationship associated with a normal mandibular arch, molar distalization is of significant value. Conventional extraoral traction has been successful in correcting class II malocclusion, either by restraining forward growth of the maxilla or by distalizing maxillary molars. However, these appliances rely partially or totally on patient cooperation.

Numerous alternative intraoral noncompliant appliances, such as pendulum,^1^ the distal jet,^2^ the K-loop molar distalizer,^3^ double loop NiTi^4^ and C space regainer^5^ have been developed, and many well-documented studies have substantiated their effects. These appliances have drawbacks of anchor loss, proclination of the maxillary incisors, tipping of the maxillary molars and difficulty in keeping the molars in position following distal movements. Space is easier to gain in the maxillary arch^6,7^ than in the mandible because of increased trabecular structure of supporting bone and increased anchorage afforded by palatal vault. Table 1^1-3,8^ shows the applications of pendulum.

In this article, a case is presented, in which maxillary molar distalization was carried out using pendulum distalization appliances. In this case, maxillary molar distalization was effective and efficient in correcting the borderline class II malocclusion.

**Table 1:** **Table 1:** When to use pendulum

*Indications of distalization*
1. Class II or end-on molar relationship
2. Mixed or permanent dentition
3. Mild to moderate crowding in maxillary arch
4. Hypodivergent or average growth pattern
5. Well-aligned teeth or mild crowding in mandibular arch
6. Straight profile
7. Functional - normal TMJ
8. Skeletal class I pattern
9. Normal/short lower face height
10. Loss of arch length due to premature loss of second deciduous molar^8^
*Contraindications*
1. Temporomandibular joint disorder
2. Class II skeletal jaw base
3. Skeletal open bite and dental open bite/shallow bite
4. Excess lower face height
5. Dental: Class I or III molar relation

## CASE REPORT

**Figs 1A to F:**
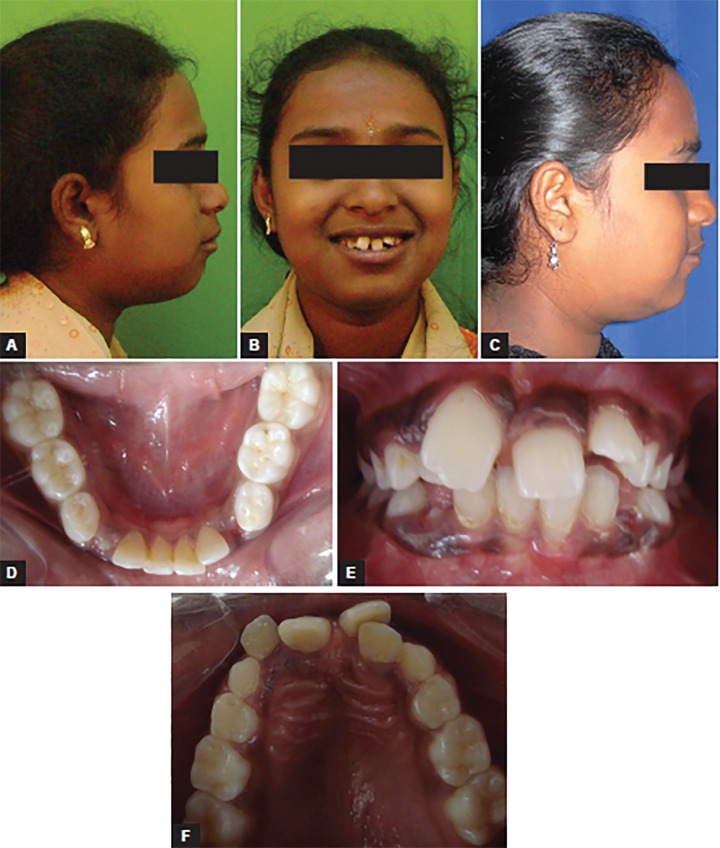
Pretreatment extraoral and intraoral photographs

**Figs 2A to D:**
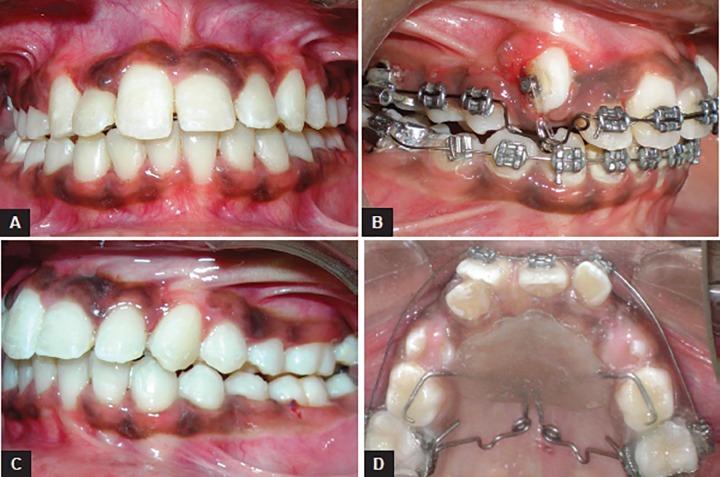
Bonded pendulum appliance for distalization of maxillary molars

**Figs 3A to E:**
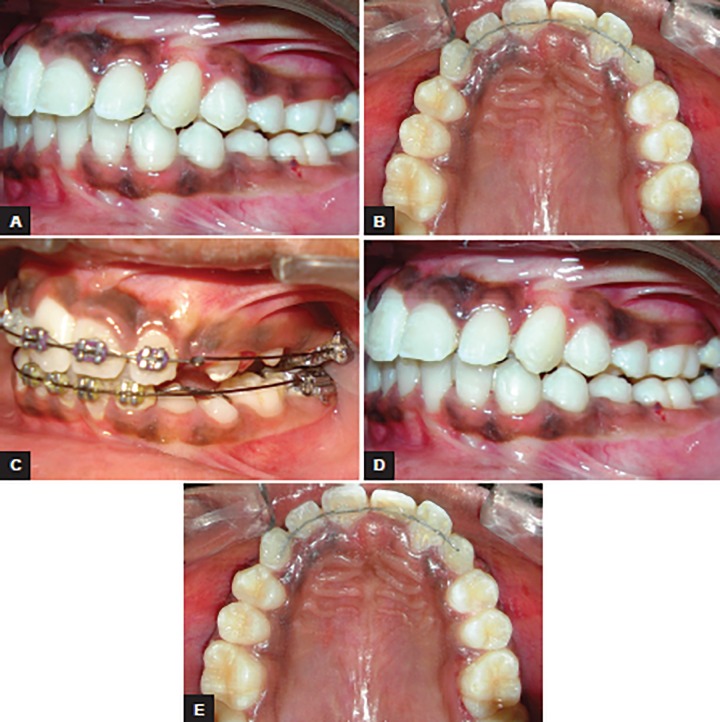
Postdistalization showing space gain

**Fig. 4:**
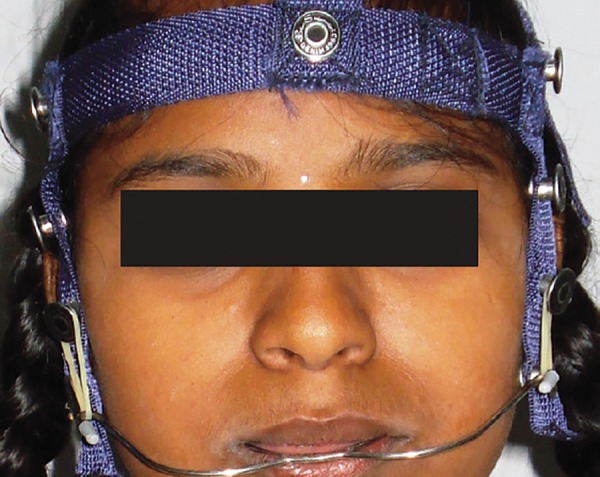
Postdistalization stabilization with headgear

**Figs 5A to C:**
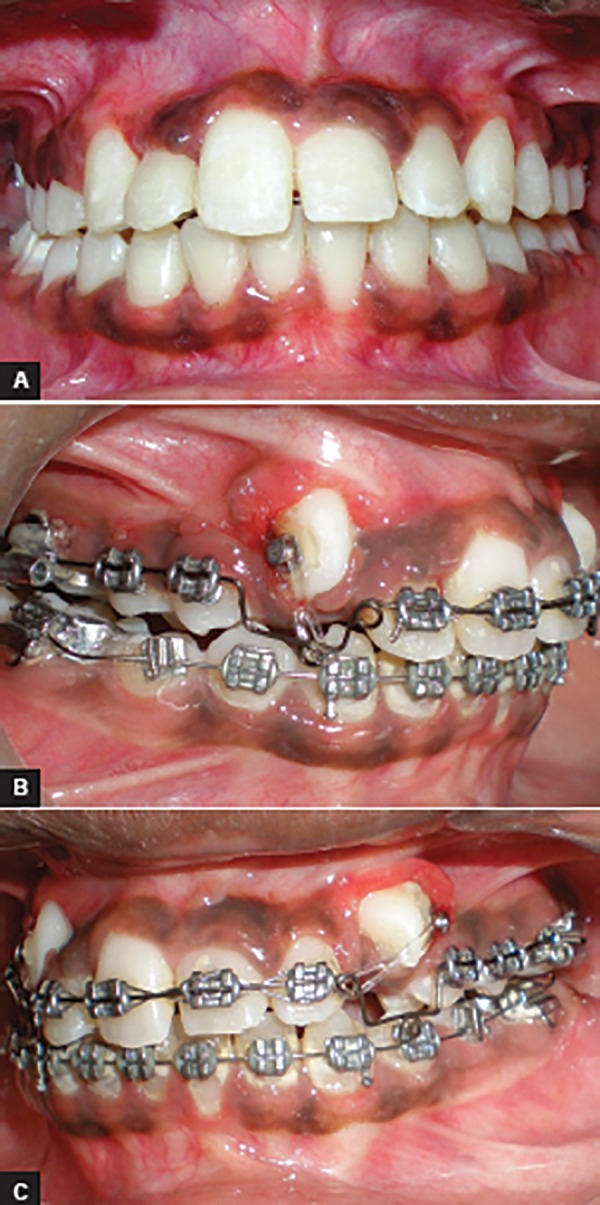
Impacted canine correction on 0.018 stainless steel wire with E-chain

**Figs 6A to E:**
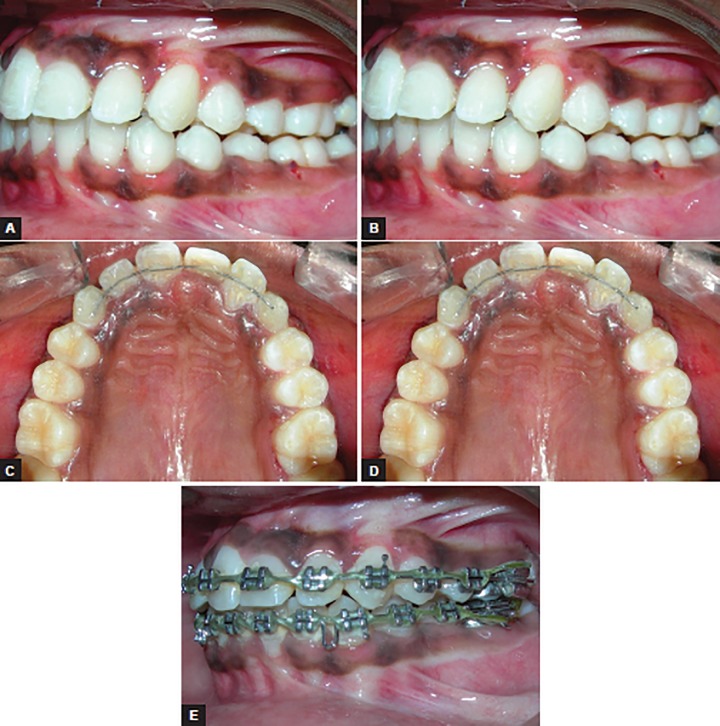
Torque correction with 0.019 × 0.025 stainless steel wire

**Figs 7A to G:**
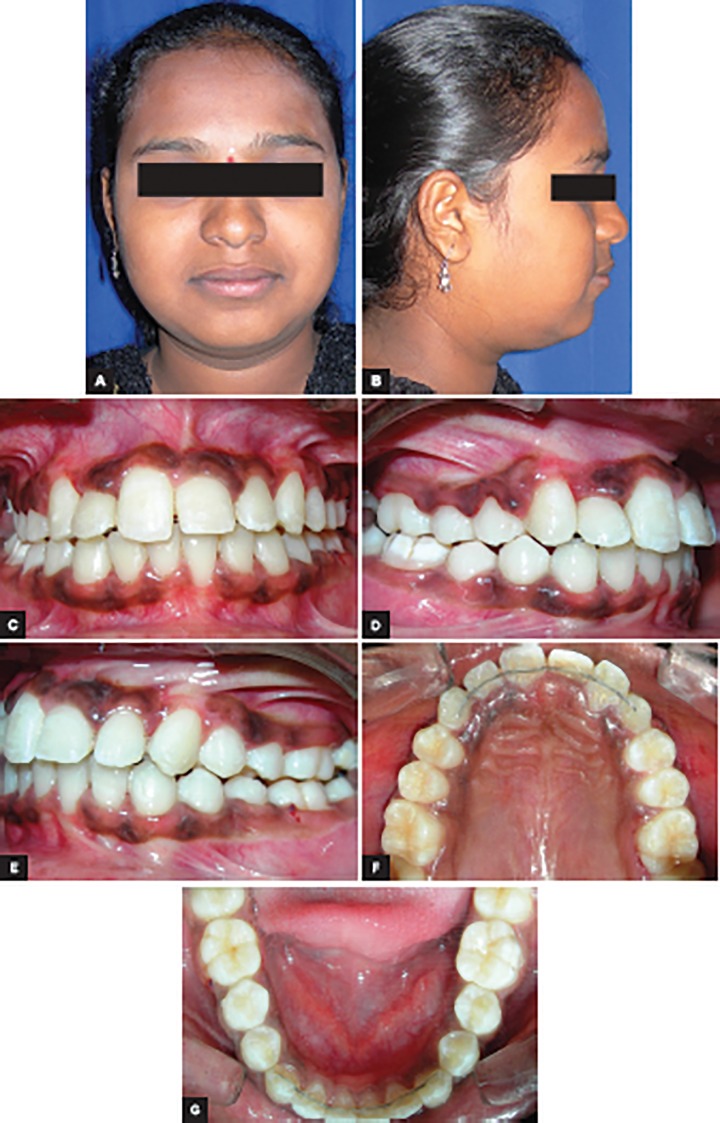
Posttreatment extraoral and intraoral photographs

A 10-year-old girl presented with the chief complaint of crowded teeth. She was in mixed dentition stage, had convex profile with average Frankfurt mandibular plane angle, class II (end-on) molar relation, upper and lower anterior crowding along with deviated midline (Fig. 1). Pendulum appliance was used in this case to correct the class II malocclusion (Fig. 2). Bonded pendulum appliance was designed to take support from deciduous molar for anchorage. With pendulum appliances, as with almost all compliance-free appliances for molar distalization described to date, the anchorage block consists of a nance palatal button and anchoring teeth in the same dental arch. The acrylic button fits tightly against the palatal mucosa in the region of the palatal rugae and is linked to the teeth with occlusally bonded onlays. After placement of the preactivated pendulum springs, the anchorage unit is designed to counteract the reactive forces and moments. Maxillary molar distalization was completed in 4 months (Fig. 3). Space gained in each side was 4 mm. The rate of distalization was almost 1 mm per month. Stabilization of class II molar relation with headgear and nance button was done to hold the gained space (Fig. 4). Postdistalization headgear was also given for uprighting of molars. Bilaterally maxillary canine impaction was treated after surgical exposure. Extrusion of canine was done on 0.018 stainless steel wire with E-chain as seen in Figure 5; 0.018 stainless steel wire with helices was made to apply the force and sound biomechanics. After canine alignment in the arch, 0.019 × 0025 stainless steel wire was used for torque correction (Fig. 6). Finally, good dentoalveolar changes and occlusion were achieved (Fig. 7).

## DISCUSSION

Distalization of maxillary molars is indicated for correction of class II dental malocclusions and for space gaining in cases of space deficiency. The ideal treatment with an intraoral fixed appliance for molar distalization should fulfill the following requirements: Patient compliance, acceptable esthetics, comfort and minimum anterior anchor loss (as evidenced by inclination of incisors). There should be bodily movement of the molars to avoid undesirable effects and unstable outcomes, and minimum time required during sessions for placement and activations. To achieve this, many devices have been suggested. Ghosh and Nanda in 1996 reported that the pendulum appliance is a reliable method for distalizing maxillary molars at the expense of moderate anchorage loss. Byloff and Darendeliler and Byloff showed that the pendulum appliance moved molars distally without creating bite opening, but the molars did tend to tip. Hilgers^1^ had shown that when the appliance is placed before the eruption of the second molars, two-thirds of the space gained is by molar distalization and one-third is experienced as forward shift of the anterior teeth. Pendulum appliances have several advantages which include cost-effectiveness and chair side activation.^9^ It should be remembered that patient selection for a particular method of distalization is of utmost importance and should not be overlooked.^10^

It is not right to treat a condition without adequate knowledge and understanding.^11^ When to treat and when to refer to an orthodontist should be based on honest appraisal of skill levels and preferences of treatment. Pedodontist is accountable with the decision to treat or refer. Such decisions are aimed at patient benefit since he/she should receive the correct treatment.

Using intraoral appliances, maxillary molars can routinely be moved distally with little or no patient cooperation. A distal movement rate of approximately 1 mm per month of the first molar’s crowns has been reported, but there is marked individual variation.^1-6^ One factor that influences the movement rate is the type of movement and another factor is the timing of treatment. Usually faster movement occurs when the molars are tipped, whereas bodily movement takes a longer time. A favorable time to move molars distally appears to be in the mixed dentition before the eruption of the second molars.^12^ The reason why it is more effective to move the maxillary first molars distally before the second molars have erupted is that there is one more tooth, and thus, a larger area of root surface to be moved when the second molars have erupted. Conceivably, this also implies that the strain on the anchorage teeth will increase when the first and second molars are moved simultaneously. Thus, the anchorage loss (forward movement of the maxillary incisors) will be lower if the molars are moved before eruption of the second molars. Even if the anchorage loss can be corrected with modest intervention, the amount of lower anchorage loss will result in less time-consuming correction.^13^

## CONCLUSION

Hence, to conclude, in our day-to-day practice, we come across many cases in which a class II div 1 malocclusion is developing due to the mesial drifting of the maxillary permanent first molars. This mesial drift could be the result of the loss of tooth material due to caries, the premature exfoliation/extraction of the deciduous molars or the ectopic eruption of the maxillary permanent first molars. Thus, the developing class II div 1 malocclusion can be successfully intercepted and corrected in the mixed dentition period by molar distalization.
